# Validation of the Tetracycline Regulatable Gene Expression System for the Study of the Pathogenesis of Infectious Disease

**DOI:** 10.1371/journal.pone.0020449

**Published:** 2011-05-25

**Authors:** Ashok K. Chaturvedi, Anna L. Lazzell, Stephen P. Saville, Floyd L. Wormley, Carlos Monteagudo, Jose L. Lopez-Ribot

**Affiliations:** 1 Department of Biology and South Texas Center for Emerging Infectious Diseases, University of Texas at San Antonio, Texas, United States of America; 2 Departmento de Patología, Facultad de Medicina y Odontología, Universidad de Valencia, Valencia, Spain; Louisiana State University, United States of America

## Abstract

Understanding the pathogenesis of infectious disease requires the examination and successful integration of parameters related to both microbial virulence and host responses. As a practical and powerful method to control microbial gene expression, including *in vivo*, the tetracycline-regulatable system has recently gained the favor of many investigative groups. However, some immunomodulatory effects of the tetracyclines, including doxycycline, could potentially limit its use to evaluate host responses during infection. Here we have used a well-established murine model of disseminated candidiasis, which is highly dependent on both the virulence displayed by the fungal cells and on the host immune status, to validate the use of this system. We demonstrate that the pathogenesis of the wild type *C. albicans* CAF2-1 strain, which does not contain any tet-regulatable element, is not affected by the presence of doxycycline. Moreover levels of key cytokines, chemokines and many other biomarkers, as determined by multi-analyte profiling, remain essentially unaltered by the presence of the antibiotic during infection. Our results indicate that the levels of doxycycline needed to control the tetracycline regulatable promoter gene expression system have no detectable effect on global host responses during candidiasis. Because tet-regulatable systems are now being increasingly used in a variety of pathogenic microorganisms, these observations have wide implications in the field of infectious diseases.

## Introduction

The pathogenesis of infectious disease is complex and results from a very delicate balance between intrinsic virulence attributes displayed by the invading microorganism and the host responses that try to counteract this attack, and it is this interaction which ultimately determines the outcome of infection [1], [2], [3]. However, most reports on this topic are uni-dimensional, either focusing solely on virulence attributes (“the microbiologist perspective”) or host immune responses (“the immunologist perspective”), but very rarely on both [4]. With the advent of the molecular era, a variety of novel tools, mostly gene disruption techniques, have been used for the identification and characterization of virulence determinants in pathogenic microorganisms, basically following Koch's molecular postulates of infectious disease [5]. Most recently, the tet-regulatable system for control of gene expression [6], [7] has been adapted and used in a variety of microorganisms, including fungi, parasites and even bacteria [8], [9], [10], [11], [12], [13]. This system contains a transactivator and a responsive promoter. The promoter is active in the absence of tetracycline or one of its derivatives (most commonly doxycycline is used), and the tet-regulated genes are transcribed, typically at high levels. Because of this, the system is normally referred to as “tet-off” system. On the other hand, transcription from the regulatable promoter is repressed after the addition of the antibiotic, even at relatively low levels. A complementary system, (“tet-on”) functions in an opposite fashion: it requires the antibiotic to bind to the operator sequences therefore activating transcription, although in this case higher concentrations of antibiotic are required [6]. One of the most appealing features of these systems is the ability to regulate gene expression *in vivo*, by simply adding doxycycline (or not) to an animal's drinking water, thus allowing the use of these genetically engineered microbial strains in their corresponding animal models of infection. Typically, the high specificity and affinity of the antibiotic allows for the use of doxycycline at low concentrations to confer tight control of gene expression, particularly in the case of “tet-off” systems [6].

Since the tetracycline regulatable system was developed specifically as a genetic tool to control/manipulate gene expression, it is tempting to directly ascribe the differences in pathogenesis of any such experiments to a “virulence” effect only. However, most tetracyclines display a number of non-antibiotic effects, including anti-oxidant, anti-apoptotic, anti-proteolytic, anti-angiogenic, anti-metastatic, and most importantly, overall immunomodulatory and anti-inflammatory effects [14], [15], [16], [17]. Therefore, if manifested, these effects could potentially influence the outcome of infection and complicate the interpretation of pathogenesis studies when animals are treated with these antibiotics [14], [18]. Moreover, they may also limit the usefulness of strains constructed using the tet-regulatable systems for examining host responses during infection.

Thus, it is important to validate the use of this system for the study of host immune responses in the pathogenesis of infectious disease. Here, we sought to demonstrate that doxycyline, under the standard conditions used to regulate gene expression *in vivo* does not display significant effects on global host responses during the infectious process. To this end we have used a well established murine model of hematogenously disseminated candidiasis, since, as an opportunistic pathogen, the fungus *Candida albicans* is one of the microorganisms in which this balance between virulence and host responses is best exemplified.

## Materials and Methods

All animal experimentation was conducted following the National Institutes of Health guidelines for housing and care of laboratory animals and performed in accordance with Institutional regulations after pertinent review and approval by the Institutional Animal Care and Use Committee at The University of Texas at San Antonio (Institutional Welfare Assurance number A3592-01, IACUC protocol number MU018).

### Strain and media

*C. albicans* CAF2-1 strain [19] was used for these studies. This is a URA3/ura3 heterozygous strain derived from the SC5314 used in the genome sequencing project. The fully virulent CAF2-1 strain is frequently used as a wild-type control for virulence studies, particularly in the characterization of isogenic mutant strains constructed using the URA blaster technique [19].

### **Animal** experiments

Cultures of *C. albicans* CAF2-1 strain for injection were grown overnight at 25°C in yeast extract-peptone-dextrose (YPD) medium without doxycycline. Cells were harvested by centrifugation and washed. After counting, appropriate dilutions were made and cells (in a final volume of 200 µl) were injected into the lateral tail veins of 6–8 week old female BALB/c mice. Five mice were used for each of the four experimental groups, which were as follows: two of these groups were given drinking water containing 5% sucrose and 2 mg/ml doxycycline starting three days prior to infection (this is the antibiotic concentration and regimen regularly used to control gene expression when using tet-regulatable strains, [12], [20]), whereas the other two groups of animals were given drinking water with sucrose (5%) only; for each condition (addition or omission of antibiotic) one group of animals was infected with the *C. albicans* CAF2-1 strain via tail vein and the other group was not inoculated (served as uninfected controls). The infection experiments were also repeated using a different infecting inoculum for survival assessment. Another four parallel groups (same conditions as above regarding infection and antibiotic treatment) of mice were included for a time-scheduled sacrifice at 3 days post-infection. In these animals the brain, spleen, and kidneys were removed for the determination of fungal burden, as well as for analyses of host responses (see below). One kidney from each animal was put aside for histological analysis; these were fixed in 10% buffered formalin, embedded in paraffin, and thin tissue slices were removed and stained with Grocott-Gomori methenamine-silver (GMS) for fungal elements and with hematoxylin-eosin (HE) staining for tissue pathology. Fungal organ burdens were determined from the remaining samples by weighing and homogenizing the tissues (as below) and then plating aliquots onto solid YPD media to determine viable colony forming units (CFU).

### Analyses of host responses

All analyses of host responses were performed using organs and serum samples recovered at time-scheduled sacrifices three days post-infection (or at the exact same time for matched uninfected animals). After necropsy, 300 µl PBS with complete protease inhibitor cocktail (Roche Diagnostics GmbH, Germany) were used for homogenization of tissues (kidney and spleen), and centrifuged at 12,000 rpm for 5 min at 4°C. The sample supernatants were collected and stored at −80°C until further analyses. Blood was obtained via cardiac puncture, placed in serum separator tubes, centrifuged and serum was stored at −80°C until analyzed. For individual sample analysis of each mouse from each group, Multi-plex cytokine and chemokine array analysis was performed using the Bio-Plex Pro™ protein multi-array system (BIO-RAD Lab, Inc. Hercules, Calif.); a mouse 23-plex assay was used according to the recommendations of the manufacturer. Proteins assayed included interleukin-1α (IL-1α), IL-1β, IL-2, IL-3, IL-4, IL-5, IL-6, IL-9, IL-10, IL-12 (p40), IL-12 (p70), IL-13, IL-17, eotaxin, granulocyte colony stimulating factor (G-CSF), granulocyte monocyte colony stimulating factor (GM-CSF), interferon-γ (IFN-γ), KC (keratinocyte-derived chemokine, a potent neutrophil chemoattractant which is the functional mouse homolog of human IL-8), monocyte chemotactic protein 1 (MCP-1), macrophage inflammatory protein-1α (MIP-1α), MIP-1β, RANTES and tumor necrosis factor-α (TNF-α).

In addition, pooled samples from each group of mice containing equal amounts of tissue homogenates (from kidney and spleen) or serum from each animal in the same experimental group were submitted to Rules Based Medicine for multianalyte profiling using the Luminex®-based Multi-Analyte Profile (MAP) technology platform (Rules Based Medicine, Austin, Texas). Analytes measured included apolipoprotein (Apo) A1, CD40, CD40 ligand, C-reactive protein (CRP), epidermal growth factor, endothelin-1, eotaxin, factor VII, fibroblast growth factor (FGF)–9, FGF-basic, fibrinogen, granulocyte chemotactic protein–2, granulocyte macrophage-colony–stimulating factor, glutathione S-transferase α, haptoglobin, interferon-gamma, immunoglobulin A (IgA), IL-1, IL-11, IL-12p70, IL-17, IL-18, IL-1α, IL-1β, IL-2, IL-3, IL-4, IL-5, IL-6, IL-7, 10-kDa interferon-gamma-inducible protein (IP-10), the neutrophil chemoattractant KC/GRO alpha, leukemia inhibitory factor (LIF), lymphotactin, MCP-1, MCP-3, MCP-5, macrophage-colony-stimulating factor, macrophage-derived chemokine, MIP–1α, MIP-1β, MIP-1γ, MIP-2, MIP-3β, matrix metalloproteinase–9 (MMP-9), myeloperoxidase (MPO), myoglobin, oncostatin M, RANTES, serum amyloid P-component (SAP), stem cell factor, serum glutamic-oxaloacetic transaminase (SGOT), tissue inhibitor of metalloproteinase type-1 (TIMP-1), tissue factor, tumor necrosis factor alpha (TNF-α), thrombopoietin (TPO), vascular cell adhesion molecule 1 (VCAM-1), vascular endothelial cell growth factor (VEGF), and von Willebrand factor (vWF). Analyses were performed in a Luminex 100 instrument (Luminex, Austin, Texas).

### Statistical analysis

Survival data and differences between groups were analyzed using the Kaplan-Meier logrank test. The Mann-Whitney test was used to determine statistical significance for differences in CFU data. A Student's t-test was used to compare levels of cytokines and chemokines between the corresponding samples from doxycycline treated and untreated groups of animals. P<0.05 was considered statistically significant. Analyses were performed using Prism by GraphPad Prism (GraphPad Software Inc., San Diego, CA).

## Results

### The course of hematogenously disseminated candidiasis after infection with a wild type *Candida albicans* strain is not affected by the presence of doxycycyline

**Figure 1:**
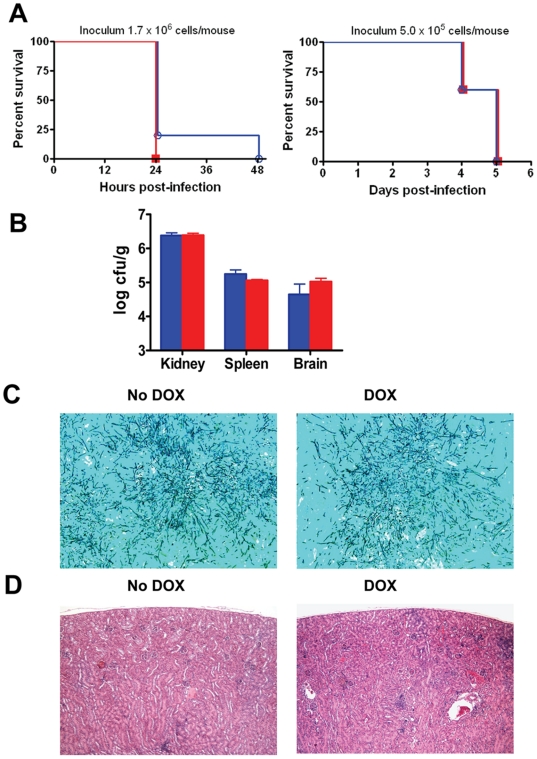
Doxycycline does not affect the course of hematogenously disseminated candidiasis in mice infected with the *C. albicans* CAF2-1 strain. Panel A, Survival curves for groups of mice infected with the *C. albicans* CAF2-1 strain in both the presence (red) or absence (blue) of doxycycline at two different infecting inocula. No statistically significant differences were detected between doxycycline-treated and untreated animals. Panel B, Organ fungal burdens at time of sacrifice (three days post-infection) for two different groups of mice challenged with 5×10^5^ cells of *C. albicans* CAF2-1 strain in the presence (red) or absence (blue) of doxycycline. Results are expressed as geometric means and standard deviations for log CFU/g values. For all organs analyzed, no statistically significant differences were detected between doxycycline-treated and untreated mice. Panel C, Morphology of *C. albicans* CAF2-1 cells present in kidneys retrieved from doxycycline-treated and untreated mice as revealed by GMS staining. The morphology of fungal elements in tissues, mostly filamentous, was indistinguishable regardless of antibiotic treatment. Magnification is x100. Panel D, Histopathological analysis of kidneys retrieved from mice after infection with *C. albicans* strain CAF2-1 strain in the presence or absence of doxycycline as revealed by H & E staining, displaying small, mostly cortical lesions irrespective of antibiotic treatment. Magnification is x40. DOX: doxycycline.

For all experiments we used the *C. albicans* CAF2-1 strain in the murine model of hematogenosuly disseminated candidiasis in which, both virulence attributes and host immune responses play important roles. This strain is well characterized as it serves as the wild type parental strain for the construction of a majority of *C. albicans* deletion mutant strains [19]. As such, the CAF2-1 strain does not contain any tetracycline regulatable element. Thus, in this strain presence or absence of doxycycline does not regulate gene expression, and should not affect the intrinsic virulence of this strain. It follows that any differences on the overall pathogenicity observed in the presence of the antibiotic using this animal model should be strictly due to an effect on host responses. As seen in Figure 1, by all assessment methods including survival, fungal organ burden and histology, the pathogenicity of the *C. albicans* CAF2-1 strain in this animal model was unaltered by the presence of doxycycline. Two different infecting inocula were tested to monitor the differences in mice survival, and in both cases the corresponding survival curves for doxycycline-treated (2 mg/ml in drinking water containing 5% sucrose) and untreated animals were almost identical (Fig 1A), with no statistically significant differences detected between the groups. The same was true for fungal burden, as organ loads in kidney, spleen and brain were similar, irrespective of antibiotic treatment (Figure 1B). As expected, the morphology of fungal cells in infected tissues was also unaffected by the presence of doxycycline (Fig 1C). Perhaps most important from the point of view of host responses, histopathological findings were virtually identical for both groups of animals (treated and untreated), as shown in Fig 1D. Overall, these results demonstrate a lack of effect of the antibiotic on virulence mechanisms (as anticipated) and, most importantly for the purpose of the current study, also point to a lack of antibiotic effect on the overall pathogenesis of candidiasis.

### Lack of effect of doxycycline on host responses during disseminated candidiasis

**Figure 2:**
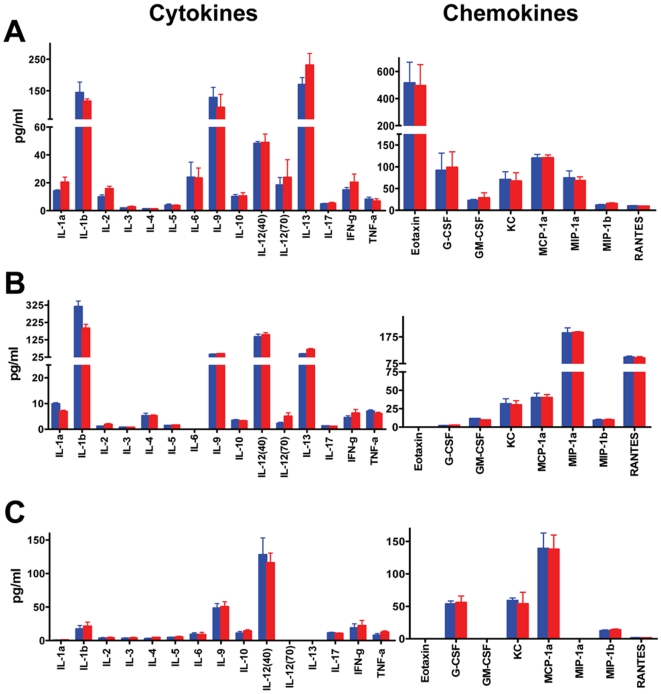
Doxycycline does not affect host immune responses during systemic candidiasis. Determination of cytokine and chemokine levels, using the Bio-Plex Pro protein multi-array system, in kidney (panel A), spleen (panel B) and serum samples (panel C) in groups of mice (n = 5 per group) three days after challenge with *C. albicans* strain CAF2-1, in the absence (blue bars) or presence (red bars) of doxycycline in the animals' drinking water. Results are presented as averages and standard deviations. No statistically significant differences were detected between doxycycline-treated and untreated animals for any of the chemokines and cytokines analyzed.

The previous set of experiments indicated a lack of an effect of doxycycline on the overall course of infection and pathogenesis of hematogenously disseminated candidiasis in mice. However, it is important to corroborate these observations by determining the levels of specific chemokines, cytokines and other markers of infection and host response in samples obtained from animals with and without the antibiotic in their water. A first level of analysis was performed using individual samples (organ homogenates and serum samples) obtained from every mouse in each group using the Bio-Plex Mouse Cytokine 23-Plex. We first demonstrated that, in the absence of infection, doxycycline does not alter basal levels of expression of these analytes (see Figure S1). Also, as shown in Figure 2, levels of cytokines, chemokines and growth factors in kidney and spleen homogenates, as well as in serum samples after infection with the *C. albicans* CAF2-1 strain, were virtually indistinguishable between doxycyline-treated and untreated groups of mice.

**Figure 3:**
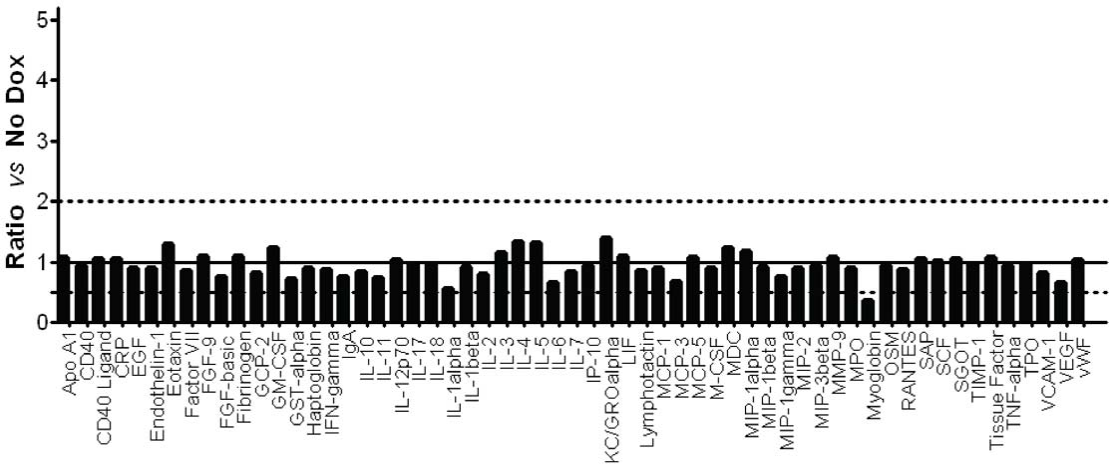
Doxycycline does not affect an expanded number of host analytes and biomarkers during systemic candidiasis Multiplex analysis using the mouse multi-analyte profiling (MAP, Rules-Based Medicine) of pooled kidney tissue homogenates obtained from a group of doxycycline-treated mice (n = 5) three days post-infection with *C. albicans* CAF2-1 strain as compared to doxycycline-untreated (control) animals. Comparative values are expressed as Ratio *vs* Control (infected in the absence of doxycycline, sacrificed at the very same time), which is arbitrarily assigned a value of 1 for each analyte and indicated by the solid grid line along the y axis. The dotted grid lines along the y axis indicate ratios of 0.5 and 2, set up arbitrarily; any analyte for which the corresponding value was below or above this range was considered to be affected by the antibiotic.

A more comprehensive analysis of the global host response in the different groups of mice was performed by multianalyte profiling using the Rules Based Medicine's mouse MAP, that allows for the concomitant examination of an extended panel of broad spectrum biomarkers relevant to infection (a total of 59 analytes, including cytokines, chemokines, growth factors, acute-phase reactants and other metabolites). Figure 3 shows the results of multianalyte profiling analyses for kidney samples (the kidney is the main target of infection) obtained from doxycycline-treated mice 3 days after infection with *C. albicans* CAF2-1 strain, compared to animals infected in the absence of the antibiotic. This direct comparison revealed no major differences in levels of the multiple analytes between doxycycline-treated versus untreated mice, thus corroborating that the antibiotic has a minimal (if any) effect on host responses under the conditions used. Similar results were obtained in a comparison between the doxycycline-treated and untreated uninfected animals, further demonstrating that basal levels of these biomarkers in the absence of infection are unaffected by doxycycline treatment (see Figure S2). The absolute values for the different analytes in each group of mice are provided in Table S1. Results from similar analyses for spleen homogenates and serum samples also indicated a global lack of effect of antibiotic treatment on systemic host responses, both with and without infection (data not shown).

## Discussion

In order to validate the tet-regulatable system we have used a murine model of hematogenously disseminated candidiasis. *C. albicans* is a formidable opportunistic human pathogen: in compromised hosts this fungus can cause disease, ranging from superficial to life-threatening invasive candidiasis [21]. In fact, mostly due to the progress of modern medicine and an expanding population of severely immunodepressed patients, disseminated candidiasis now represents the third to fourth most common nosocomial infection worldwide with, most importantly, unacceptably high morbidity and mortality rates [22], [23], [24], [25]. In this animal model of infection, both virulence attributes and host immune responses play important roles [26], [27], [28], [29], [30], which makes this model an ideal one for the specific purpose of this study. The infection reasonably mimics the progression of disease observed in humans, and faithfully recapitulates the main events associated with systemic disease; including circulation of cells through the vasculature, extravasation, dissemination to distal sites, establishment of deep seated infection, and sepsis [31], [32], [33].

For the direct assessment of specific virulence attributes in *C. albicans*, most studies have used the URA blaster [19] or similar gene disruption techniques to generate knock-out mutant strains and directly compare the virulence of the corresponding gene deletion strains versus that of the parental strain. More recently, the conditional tetracycline-regulatable promoter system to control gene expression was adapted for *Candida* and used to explore several aspects of *C. albicans* pathogenicity [12], [20], [34], [35]. In the “tet-off” system, a tetracycline-controlled transactivator interacts with a responsive promoter to regulate expression of the gene of interest. Expression is controlled from outside by a tetracycline, most commonly doxycycline. The antibiotic is widely considered safe and nontoxic at the relatively low doses required for gene inactivation [6], [36]. However, some immunomodulatory effects of the tetracyclines could potentially limit its use to evaluate host responses during infection [14], [15], [16], [17]. Thus, we performed a series of experiments to validate the use of this system in the analyses of host responses during the pathogenesis of candidiasis. We demonstrate that, in stark contrast with results observed with genetically engineered tet-regulatable strains that display clearly demonstrable effects on virulence [20], [34], [35], [37], the course of infection and overall pathogenesis of the wild type *C. albicans* CAF2-1 strain is not affected by the presence or absence of doxycycline. Moreover levels of key cytokines, chemokines and many other biomarkers, as determined by multi-analyte profiling, remain essentially unaffected by the presence or absence of the antibiotic during infection, clearly indicating that doxycycline, under these conditions, does not affect host responses during the infectious process.

Taken together, our results demonstrate that doxycycline, at the standard concentrations used to regulate gene expression in the tet-off system, does not alter host responses during infection. Of course, these results should be approached with caution and may not be applicable to mucosal models of infection, where the tetracyclines' antibiotic effect may impact the normal bacterial microbiota; it may be possible to overcome this limitation, however, by using chemically modified tetracyclines and tetracycline analogues which lack antibacterial activity [38]. We also note that our observations may not necessarily extend to the so called “tet-on” system, which generally requires a higher concentration of antibiotic to activate gene expression [6], [39]. Added to the well characterized safety, pharmacodynamic, pharmacokinetic and favorable toxicological properties of these antibiotics [6], [36], these results further validate the utility of microbial strains constructed using the tet-regulatable gene expression system in pathogenesis studies and for the analyses of host responses during infection. Our results provide reassurance that under the standard conditions of doxycycline use, any observed difference in pathogenesis is overwhelmingly due to the antibiotic's control of gene expression (*i.e.* effect on virulence of the pathogen) and not due to any immunoregulatory effects exerted by the drug. Because of the increased popularity of tet-regulatable systems to control gene expression in a variety of pathogenic microorganisms, including parasites, fungi and bacteria [8], [9], [10], [13], [20], [34], and the use of these genetically engineered strains in the corresponding animal models of infection, our observations have broad applicability in the fields of Microbiology and Infectious Diseases. Moreover, they transcend these fields since tet-regulatable gene expression techniques are also being increasingly used in mamalian systems, including transgenic animal models and gene therapy applications in humans [40], [41].

## Supporting Information

Figure S1 **Determination of cytokine and chemokine basal levels, using the Bio-Plex Pro protein multi-array system, in kidney (panel A), spleen (panel B) and serum samples (panel C) from uninfected groups of mice (n = 5 per group) in the absence (blue bars) or presence (red bars) of doxycycline in their drinking water. Results are averages and standard deviations.** No statistically significant differences were detected between doxycycline-treated and untreated animals for any of the chemokines and cytokines analyzed.
(PDF)

Figure S2 **Multiplex analysis using the mouse multi-analyte profiling (MAP, Rules-Based Medicine) of pooled kidney tissue homogenates obtained from a group of doxycycline-treated mice (n = 5) in the absence of infection as compared to doxycycline-untreated animals.** Comparative values are expressed as Ratio *vs* Control (uninfected and in the absence of doxycycline, sacrificed at the very same time), which is arbitrarily assigned a value of 1 for each analyte and indicated by the solid grid line along the y axis. The dotted grid lines along the y axis indicate ratios of 0.5 and 2; any analyte for which the corresponding value was below or above this range was arbitrarily considered differentially expressed.
(PDF)

Table S1 **Absolute values for concentrations of the different analytes determined at time of sacrifice in pooled (n = 5) kidney homogenates from the different groups of mice (infected and uninfected, in the absence or presence of doxycycline-DOX), using Rules Based Medicine's Rodent MAP.**
(PDF)
