# Supplementary material for: Validation of the Tetracycline Regulatable Gene Expression System for the Study of the Pathogenesis of Infectious Disease
Source: PLoS One. 2011 May 25;6(5):e20449. doi: 10.1371/journal.pone.0020449 (PMC3102114; doi:10.1371/journal.pone.0020449)
Supplement: Figure S1 — Determination of cytokine and chemokine basal levels, using the Bio-Plex Pro protein multi-array system, in kidney (panel A), spleen (panel B) and serum samples (panel C) from uninfected groups of mice (n = 5 per group) in the absence (blue bars) or presence (red bars) of doxycycline in their drinking water. Results are averages and standard deviations. No statistically significant differences were detected between doxycycline-treated and untreated animals for any of the chemokines and cytokines analyzed. (PDF) [file pone.0020449.s001.pdf]

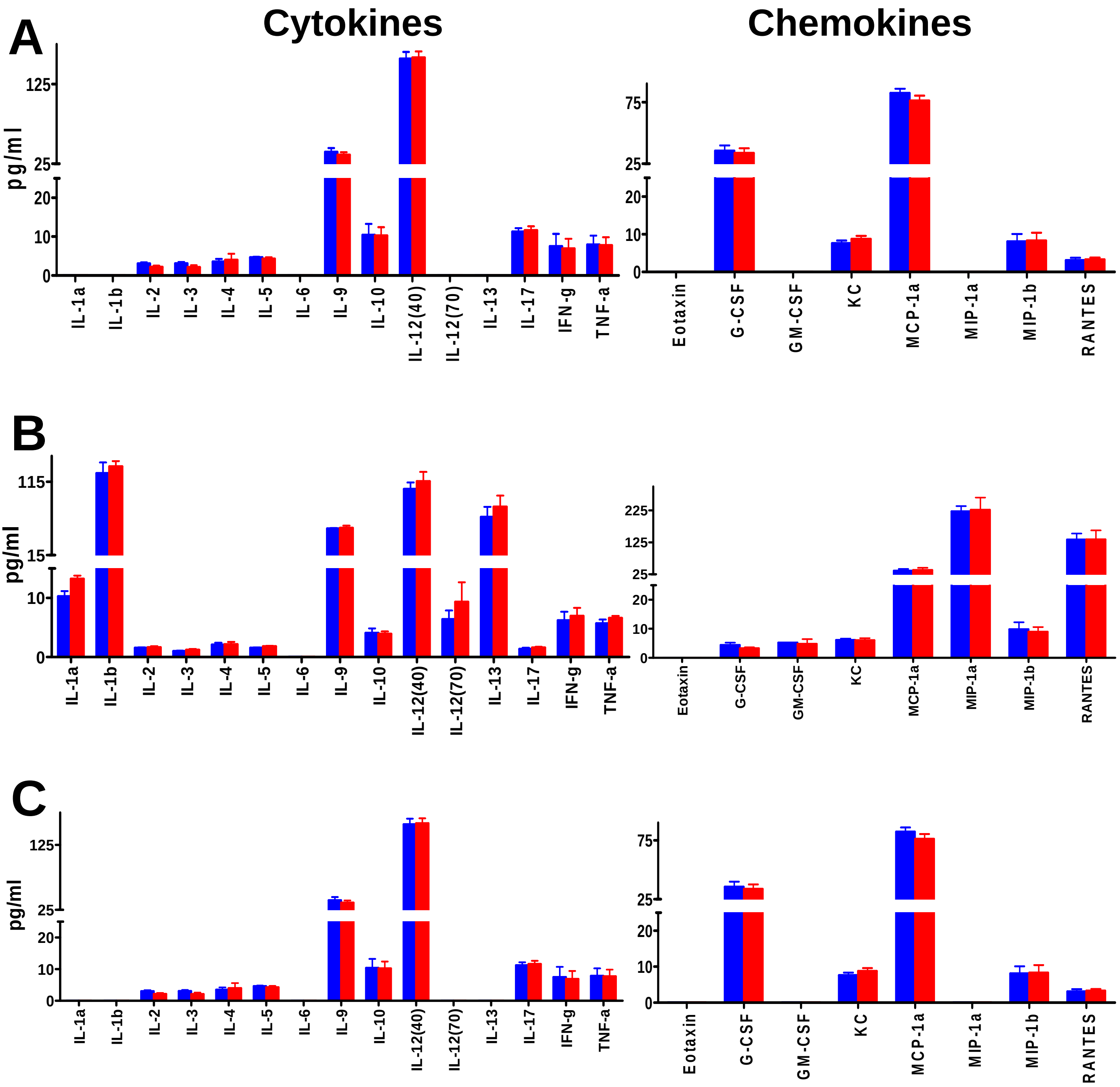

Supplementary Figure S1. Determination of cytokine and chemokine basal levels, using the Bio-Plex Pro protein multi-array system, in kidney (panel A), spleen (panel B) and serum samples (panel C) from uninfected groups of mice (n = 5 per group) in the absence (blue bars) or presence (red bars) of doxycycline in their drinking water. Results are averages and standard deviations. No statistically significant differences were detected between doxycycline-treated and untreated animals for any of the chemokines and cytokines analyzed.
