# Supplementary material for: Validation of the Tetracycline Regulatable Gene Expression System for the Study of the Pathogenesis of Infectious Disease
Source: PLoS One. 2011 May 25;6(5):e20449. doi: 10.1371/journal.pone.0020449 (PMC3102114; doi:10.1371/journal.pone.0020449)
Supplement: Figure S2 — Multiplex analysis using the mouse multi-analyte profiling (MAP, Rules-Based Medicine) of pooled kidney tissue homogenates obtained from a group of doxycycline-treated mice (n = 5) in the absence of infection as compared to doxycycline-untreated animals. Comparative values are expressed as Ratio vs Control (uninfected and in the absence of doxycycline, sacrificed at the very same time), which is arbitrarily assigned a value of 1 for each analyte and indicated by the solid grid line along the y axis. The dotted grid lines along the y axis indicate ratios of 0.5 and 2; any analyte for which the corresponding value was below or above this range was arbitrarily considered differentially expressed. (PDF) [file pone.0020449.s002.pdf]

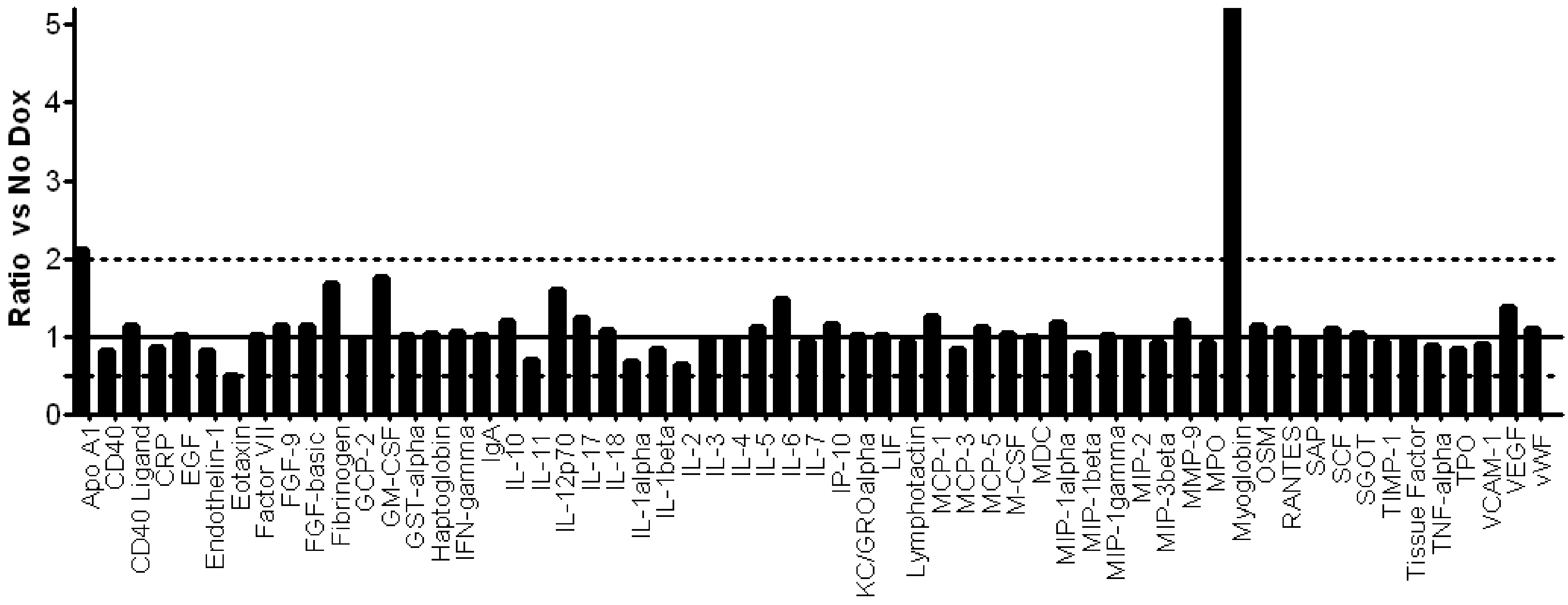

Supplementary Figure S2. Multiplex analysis using the mouse multi-analyte profiling (MAP, Rules-Based Medicine) of pooled kidney tissue homogenates obtained from a group of doxycycline-treated mice (n = 5) in the absence of infection as compared to doxycycline-untreated animals. Comparative values are expressed as Ratio *vs* Control (uninfected and in the absence of doxycycline, sacrificed at the very same time), which is arbitrarily assigned a value of 1 for each analyte and indicated by the solid grid line along the y axis. The dotted grid lines along the y axis indicate ratios of 0.5 and 2; any analyte for which the corresponding value was below or above this range was arbitrarily considered differentially expressed.
