# Supplementary material for: Validation of the Tetracycline Regulatable Gene Expression System for the Study of the Pathogenesis of Infectious Disease
Source: PLoS One. 2011 May 25;6(5):e20449. doi: 10.1371/journal.pone.0020449 (PMC3102114; doi:10.1371/journal.pone.0020449)
Supplement: Table S1 — Absolute values for concentrations of the different analytes determined at time of sacrifice in pooled (n = 5) kidney homogenates from the different groups of mice (infected and uninfected, in the absence or presence of doxycycline-DOX), using Rules Based Medicine's Rodent MAP. (PDF) [file pone.0020449.s003.pdf]

Supplementary Table 1. Absolute values for concentrations of the different analytes determined at time of sacrifice in pooled (n = 5) kidney homogenates from the different groups of mice (infected and unifected, in the absence or presence of doxycycline - DOX), using Rules Based Medicine's Rodent MAP.

	Uninfected no DOX	Uninfected plus DOX	Infected no DOX	Infected plus DOX		LDD (Homogenates) (LDD, least detectable dose)
**Apo A1 (Apolipoprotein A1)**	5.4	11	6.7	7.2	µg/mL	*0.21*
**CD40**	515	414	687	637	pg/mL	*2.4*
**CD40 Ligand**	344	387	471	493	pg/mL	*18*
**CRP (C Reactive Protein)**	0.13	0.11	0.28	0.29	µg/mL	*0.0042*
**EGF (Epidermal Growth Factor)**	7.0	7.0	11	9.3	pg/mL	*7.8*
**Endothelin-1**	31	25	33	29	pg/mL	*13*
**Eotaxin**	27	13	28	35	pg/mL	*2.5*
**Factor VII**	2.7	2.6	3.1	2.6	ng/mL	*0.19*
**FGF-9 (Fibroblast Growth Factor-9)**	5.2	5.8	5.5	5.9	ng/mL	*0.20*
**FGF-basic (Fibroblast Growth Factor-basic)**	17	19	19	14	ng/mL	*0.12*
**Fibrinogen**	156	260	428	466	µg/mL	*0.85*
**GCP-2 (Granulocyte Chemotactic Protein-2)**	0.10	0.096	0.12	0.099	ng/mL	*0.0049*
**GM-CSF (Granulocyte Macrophage-Colony Stimulating Factor)**	0.46	0.80	1.2	1.4	pg/mL	*1.7*
**GST-alpha (Glutathione S-Transferase alpha)**	0.27	0.27	0.28	0.19	ng/mL	*0.084*
**Haptoglobin**	0.69	0.71	3.8	3.3	µg/mL	*0.013*
**IFN-alpha (Interferon-alpha)**	14	14	17	15	pg/mL	*14*
**IgA (Immunoglobulin A)**	1.2	1.2	1.3	0.95	µg/mL	*0.038*
**IL-10 (Interleukin-10)**	1380	1640	1110	900	pg/mL	*22*
**IL-11 (Interleukin-11)**	16	11	43	31	pg/mL	*17*
**IL-12p70 (Interleukin-12p70)**	0.37	0.57	0.35	0.36	ng/mL	*0.11*
**IL-17 (Interleukin-17)**	0.0065	0.0080	0.0084	0.0080	ng/mL	*0.030*
**IL-18 (Interleukin-18)**	0.85	0.91	1.00	0.94	ng/mL	*0.13*
**IL-1alpha (Interleukin-1alpha)**	123	82	261	142	pg/mL	*9.0*
**IL-1beta (Interleukin-1beta)**	1.00	0.83	1.7	1.5	ng/mL	*0.089*

**IL-2 (Interleukin-2)**	76	47	71	56	pg/mL	*13*
**IL-3 (Interleukin-3)**	2.2	2.1	4.2	4.8	pg/mL	*4.3*
**IL-4 (Interleukin-4)**	21	20	18	24	pg/mL	*15*
**IL-5 (Interleukin-5)**	0.13	0.14	0.21	0.27	ng/mL	*0.039*
**IL-6 (Interleukin-6)**	2.7	3.9	51	33	pg/mL	*2.8*
**IL-7 (Interleukin-7)**	0.11	0.10	0.12	0.10	ng/mL	*0.062*
**IP-10 (Inducible Protein-10)**	22	25	49	46	pg/mL	*8.1*
**KC/GROalpha (Melanoma Growth Stimulatory Activity Protein)**	0.0067	0.0067	0.051	0.069	ng/mL	*0.035*
**LIF (Leukemia Inhibitory Factor)**	140	141	439	470	pg/mL	*8.7*
**Lymphotactin**	37	34	55	46	pg/mL	*17*
**MCP-1 (Monocyte Chemoattractant Protein-1)**	15	18	47	41	pg/mL	*3.4*
**MCP-3 (Monocyte Chemoattractant Protein-3)**	47	39	68	46	pg/mL	*6.3*
**MCP-5 (Monocyte Chemoattractant Protein-5)**	8.2	9.0	30	32	pg/mL	*9.3*
**M-CSF (Macrophage-Colony Stimulating Factor)**	0.61	0.62	0.79	0.69	ng/mL	*0.0036*
**MDC (Macrophage-Derived Chemokine)**	42	41	54	65	pg/mL	*4.4*
**MIP-1alpha (Macrophage Inflammatory Protein-1alpha)**	0.15	0.18	0.15	0.18	ng/mL	*0.045*
**MIP-1beta (Macrophage Inflammatory Protein-1beta)**	33	25	60	54	pg/mL	*16*
**MIP-1gamma (Macrophage Inflammatory Protein-1gamma)**	1.7	1.7	2.4	1.7	ng/mL	*0.015*
**MIP-2 (Macrophage Inflammatory Protein-2)**	5.2	5.0	55	48	pg/mL	*1.4*
**MIP-3beta (Macrophage Inflammatory Protein-3beta)**	0.22	0.20	0.24	0.23	ng/mL	*0.093*
**MMP-9 (Matrix Metalloproteinase-9)**	1.5	1.7	3.3	3.5	ng/mL	*0.10*
**MPO (Myeloperoxidase)**	3.5	3.1	26	23	ng/mL	*0.19*
**Myoglobin**	5.7	33	12	4.1	ng/mL	*0.24*
**OSM (Oncostatin M)**	0.12	0.14	0.12	0.11	ng/mL	*0.026*
**RANTES**	1.6	1.7	3.2	2.8	pg/mL	*9.6*
**SAP (Serum Amyloid P)**	0.67	0.64	0.88	0.93	µg/mL	*0.027*
**SCF (Stem Cell Factor)**	1230	1330	1200	1210	pg/mL	*15*
**SGOT (Serum Glutamic-Oxaloacetic Transaminase)**	12	12	13	13	µg/mL	*0.37*
**TIMP-1 (Tissue Inhibitor of Metalloproteinase Type-1)**	0.18	0.17	1.4	1.3	ng/mL	*0.036*
**Tissue Factor**	0.98	0.93	1.1	1.2	ng/mL	*0.10*

**TNF-alpha (Tumor Necrosis Factor-alpha)**	0.052	0.045	0.062	0.057	ng/mL	*0.027*
**TPO (Thrombopoietin)**	2.7	2.2	3.5	3.3	ng/mL	*0.53*
**VCAM-1 (Vascular Cell Adhesion Molecule-1)**	71	62	116	94	ng/mL	*0.19*
**VEGF (Vascular Endothelial Cell Growth Factor)**	234	319	250	158	pg/mL	*7.6*
**vWF (von Willebrand Factor)**	4.3	4.7	5.5	5.6	ng/mL	*0.99*
